# Multidimensional analysis of brain activation patterns in different motor therapies using functional near-infrared spectroscopy

**DOI:** 10.3389/fneur.2025.1656369

**Published:** 2025-11-26

**Authors:** Xiang-Ming Lin, Yi-Shan Xue, Yu-Han Liu, Rui Hong, Wan-Rong Xu, Ying Li, Ben-Guo Wang

**Affiliations:** 1 Rehabilitation Medicine Department of the Second Affiliated Hospital, School of Medicine, The Chinese University of Hong Kong, Shenzhen & Longgang District People's Hospital of Shenzhen, Shenzhen, Guangdong, China; 2 Gannan Medical University, Ganzhou, Jiangxi, China

**Keywords:** motor therapy, active movement, passive movement, motor imagery, fNIRS, cortical excitability

## Abstract

**Objective:**

This study employed functional near-infrared spectroscopy (fNIRS) to systematically compare the effects of active movement (AM), passive movement (PM), and motor imagery (MI) on sensorimotor cortex excitability across three dimensions: spatial distribution, activation intensity, and temporal dynamics, thereby revealing distinct neural mechanisms underlying different motor therapies.

**Materials and methods:**

Sixteen healthy participants performed AM, PM, and MI tasks under therapist guidance using a block design. fNIRS data covered bilateral primary motor cortex (M1), primary somatosensory cortex (S1), supplementary motor area (SMA), dorsolateral prefrontal cortex (DLPFC), and frontopolar area (FPA). Analytical metrics included: (1) Spatial features: mean *Δ*[HbO] during 0–30s time windows; (2) Activation intensity: generalized linear model (GLM)-fitted Δ[HbO] during 0–30s; (3) Temporal dynamics: slope values during 2–7 s and T-centroid values during 0–60s.

**Results:**

Spatially, MI demonstrated the most extensive activation (bilateral DLPFC, SMA, M1, and left FPA; all *p* < 0.05, FDR-corrected), followed by AM (bilateral DLPFC, M1, and left FPA), while PM showed more limited activation (bilateral DLPFC, left S1, and right FPA). In activation intensity, AM exhibited significantly stronger activation than PM and MI in DLPFC channels 27 and 29 (both *p* < 0.05, uncorrected). Temporally, AM showed steeper slopes in left DLPFC channel 27 (*F* = 10.31, *p* = 0.034, FDR-corrected), while MI demonstrated faster responses in right S1 and SMA (both *p* = 0.03, FDR-corrected), with both PM and MI responding faster than AM in left FPA (*p* = 0.03, FDR-corrected).

**Conclusion:**

These findings reveal therapy-specific neural mechanisms: MI broadly engages motor execution and cognitive control networks through mental simulation, AM predominantly activates motor execution networks with DLPFC dominance, and PM recruits sensory-attentional networks via external facilitation. The multidimensional neuroimaging evidence provides a foundation for personalized rehabilitation protocols.

## 1 Introduction

In Parkinson’s disease (PD), Alzheimer’s disease (AD), and stroke, characteristic impairments often occur in brain networks associated with cognitive and motor functions. In clinical rehabilitation practice, motor therapy serves as a cornerstone intervention for improving these functional deficits (1, 2). Traditional motor therapies primarily consist of two fundamental modalities (3): passive movement (PM), which maintains joint range of motion through external force assistance (4), and active movement (AM), which enhances muscle strength and endurance via voluntary training (5). However, as clinical experience has accumulated, researchers have recognized that single-mode therapies often fail to address the diverse needs across different rehabilitation stages, particularly in terms of inducing neuroplasticity (6). This recognition has driven the rapid development of motor imagery (MI) therapy as a novel intervention. By inducing “offline” activation of motor-related brain areas through mental simulation, MI demonstrates unique advantages in clinical rehabilitation (7). Importantly, MI can not only be used independently but also effectively combined with traditional PM and AM to form integrated training protocols (8). In stroke rehabilitation practice, therapists have systematically implemented these combined modalities with demonstrated efficacy (9). For instance, PM-MI combined training with robotic assistance during the acute phase significantly improves motor coordination (10), while robot-assisted AM-MI training during recovery effectively enhances motor function (11).

However, research on these three motor therapies still exhibits notable limitations. Firstly, existing studies lack systematic exploration from the perspective of brain network integration. More critically, the majority of these investigations are confined to single-dimensional analyses of brain activation—focusing either on spatial patterns [e.g., fMRI localization (12)] or temporal characteristics (e.g., EEG signals (13)). This fragmented approach impedes a holistic understanding of the neural mechanisms underlying different motor therapies. Of particular concern is the current absence of studies systematically dissecting brain activation features across three key dimensions—spatial distribution, temporal dynamics, and activation intensity—which severely constrains the development and optimization of precision rehabilitation protocols. To address this pivotal scientific challenge, functional near-infrared spectroscopy (fNIRS) offers a breakthrough solution. This technology combines high temporal resolution, exceptional motion tolerance, and multichannel coverage capabilities, enabling precise quantification of oxyhemoglobin (HbO) rapid dynamic changes while simultaneously monitoring activation patterns in critical brain regions such as the prefrontal and motor cortices (14, 15). These unique advantages establish fNIRS as an ideal tool for in-depth investigation of the neural mechanisms of motor therapies.

In summary, this study will innovatively employ fNIRS technology to systematically compare brain activation characteristics across three motor therapies through multidimensional monitoring of HbO’s spatial distribution, temporal dynamics, and concentration changes. For the first time, this research will (1) establish a multidimensional brain activation signature profile for different motor therapies and (2) reveal the neural mechanism differences among these therapies across three dimensions—spatial distribution, activation intensity, and temporal dynamics. These findings will theoretically deepen the understanding of motor therapies’ neural mechanisms, overcoming the limitations of traditional single-dimensional analyses. Practically, they will provide crucial clinical insights to guide the optimization of personalized rehabilitation protocols. Technically, they will highlight fNIRS’s superior spatiotemporal resolution advantages (16)—achieving “holographic” resolution of movement-induced brain activation by simultaneously capturing millisecond-level temporal resolution and centimeter-level spatial resolution. By integrating fNIRS’s multidimensional analysis with clinical rehabilitation needs, this analytical framework will establish a new paradigm for investigating motor therapy mechanisms while offering novel technological pathways and theoretical foundations to advance precision rehabilitation systems.

## 2 Materials and methods

This study enrolled 16 healthy participants (8 males/8 females, mean age 22.31 ± 1.82 years, range: 20–26) from Shenzhen Longgang District People’s Hospital. All participants were confirmed right-handed by the Edinburgh Handedness Inventory (17), with no history of neurological, somatic, or psychiatric disorders. Participants abstained from medications potentially affecting measurements and refrained from consuming caffeine or tea (which may lower cortical excitability thresholds) prior to testing. The study protocol was approved by the Medical Research Ethics Committee of The Second Affiliated Hospital, School of Medicine, The Chinese University of Hong Kong, Shenzhen & Longgang District People’s Hospital of Shenzhen (Approval: No. 2025055). The study was registered at the Chinese Clinical Trial Registry (Registration No.: CTR2500103478). Written informed consent was obtained from all participants.

### 2.1 fNIRS

The fNIRS data were collected using the BS-7000 fNIRS V1.0 system (YIRUIDE Medical Co., Wuhan, China) at a 20 Hz sampling rate with dual-wavelength near-infrared light (690 nm and 830 nm) to measure relative concentration changes of HbO and HbR through optical attenuation. The system consisted of 32 emitter and 32 detector probes forming 106 channels covering the entire cerebral cortex (frontal, parietal, temporal, and occipital lobes). The probe layout (montage) was designed in accordance with the international 10–20 system. After cap placement, the subject’s scalp surface was carefully measured along specific anatomical landmarks: from the nasion to the inion and the horizontal line connecting the left and right preauricular points, ensuring proper alignment of the Cz electrode position on the cap with its corresponding anatomical location. Prior to data acquisition, a standardized signal quality assurance procedure was implemented: optodes were adjusted and the real-time raw light intensity signals displayed on the system interface were inspected to verify and ensure optimal optode-scalp contact; the gain for each channel was automatically optimized by the system software to maintain the detected light intensity within the manufacturer’s recommended optimal range (>90%); experiments were conducted in a room with dimmed lighting to minimize ambient light interference. Concurrently, signal quality was qualitatively assessed by visually inspecting the real-time raw signals for the presence of clear cardiac pulsation patterns, stability, and the absence of saturation or disconnection. Any channels exhibiting poor quality during this preliminary check were either re-adjusted or excluded before the commencement of the formal experimental recording.

The precise correspondence between fNIRS channels and the underlying cerebral cortical regions was determined through a two-step process. First, the NirMasterV1.0 analysis software was used to perform automated spatial registration, which coregistered the optode positions, defined by the 10–20 system, to the standard Montreal Neurological Institute (MNI) brain space, thereby obtaining the MNI coordinates for each measurement channel. Second, to achieve a more refined and objective anatomical labeling, these MNI coordinates were imported into the MRicron software with the Automated Anatomical Labeling (AAL) atlas. For each channel, the brain region that accounted for the largest probabilistic overlap within the AAL atlas was identified. Our final regions of interest (ROIs) were defined based on this objective, atlas-driven classification. Since individual channels only cover partial regions of corresponding brain areas, whereas complete functional areas typically span multiple channels, we grouped channels into regions of interest (ROIs) to reflect whole-region activation patterns. Anatomical registration was performed according to the international 10–20 system to confirm channel-cortex correspondence (18, 19). Specifically, we defined: (1) Dorsolateral prefrontal cortex (DLPFC): 14 channels (left: Ch5/6/12/19/26/27/32; right: Ch36/28/29/21/15/10/11), (2) Frontopolar area (FPA): 8 channels (left: Ch1/2/7/13; right: Ch14/9/3/4), (3) Supplementary motor area (SMA): 18 channels (left: Ch38-40/50–52/62–63/73; right: Ch75/64–65/54–56/45–47), (4) Primary motor cortex (M1): 8 channels (left: Ch84/61/72/83; right: Ch86/76/66/85), (5) Primary somatosensory cortex (S1): 4 channels (left: Ch70/60; right: Ch67/78)

### 2.2 Study protocol

#### 2.2.1 Participant preparation and signal quality assurance

This study used fNIRS to examine brain activation characteristics during upper limb motor tasks. Prior to testing, examiners positioned the optode cap by aligning its center to the Cz point (intersection of intertragal line and nasion-inion midline), adjusting optode contact pressure to ensure optimal fNIRS signal quality. Participants maintained a long-leg sitting position in a quiet environment, keeping their left upper limb, torso, and lower limbs stationary while strictly avoiding head movements or speaking. Testing began after signal stabilization, with one examiner supervising movement execution and another monitoring real-time cortical activity via fNIRS.

#### 2.2.2 Experimental design and block structure

**Figure 1:**
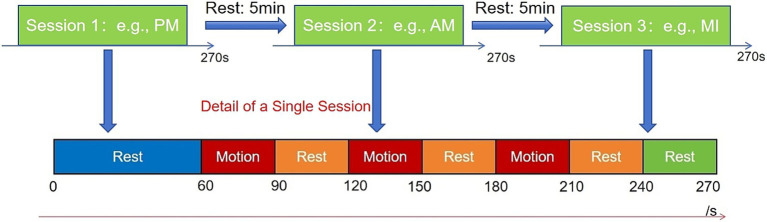
Schematic of the complete experimental protocol for the block-designed motor task. The upper section illustrates the sequence of the three experimental sessions (e.g., AM, PM, and MI), which were performed by each participant in a randomized order. A 5-min rest interval was implemented between consecutive sessions; the lower section details the structure of a single session. Each session began with a 60-s initial rest period, then contained three 30-s task execution periods (all involving repetition of the same movement), interspersed with 30-s rest periods, and concluded with a final 30-s rest period. Each session had a total duration of 270 s.

The experiment employed a block design lasting 270 s, consisting of five blocks (Figure 1): a 60 s initial rest period, three alternating 30 s task/30 s rest blocks, and a 30 s terminal rest period to allow cortical recovery. The precise timing of the block transitions was controlled by the examiner following standardized auditory cues provided by the fNIRS system. During rest intervals, participants maintained complete stillness of head, torso, and limbs while avoiding speech or facial expressions and sustaining regular breathing. Notably, each participant completed this block design three times, with each run dedicated exclusively to one of the three motor therapy conditions (AM, PM, or MI) presented in a randomized order. Each participant randomly performed three right upper limb movement paradigms (AM, PM, MI) with 5-min inter-trial intervals for signal baseline recovery.

#### 2.2.3 Motor task specification

**Figure 2:**
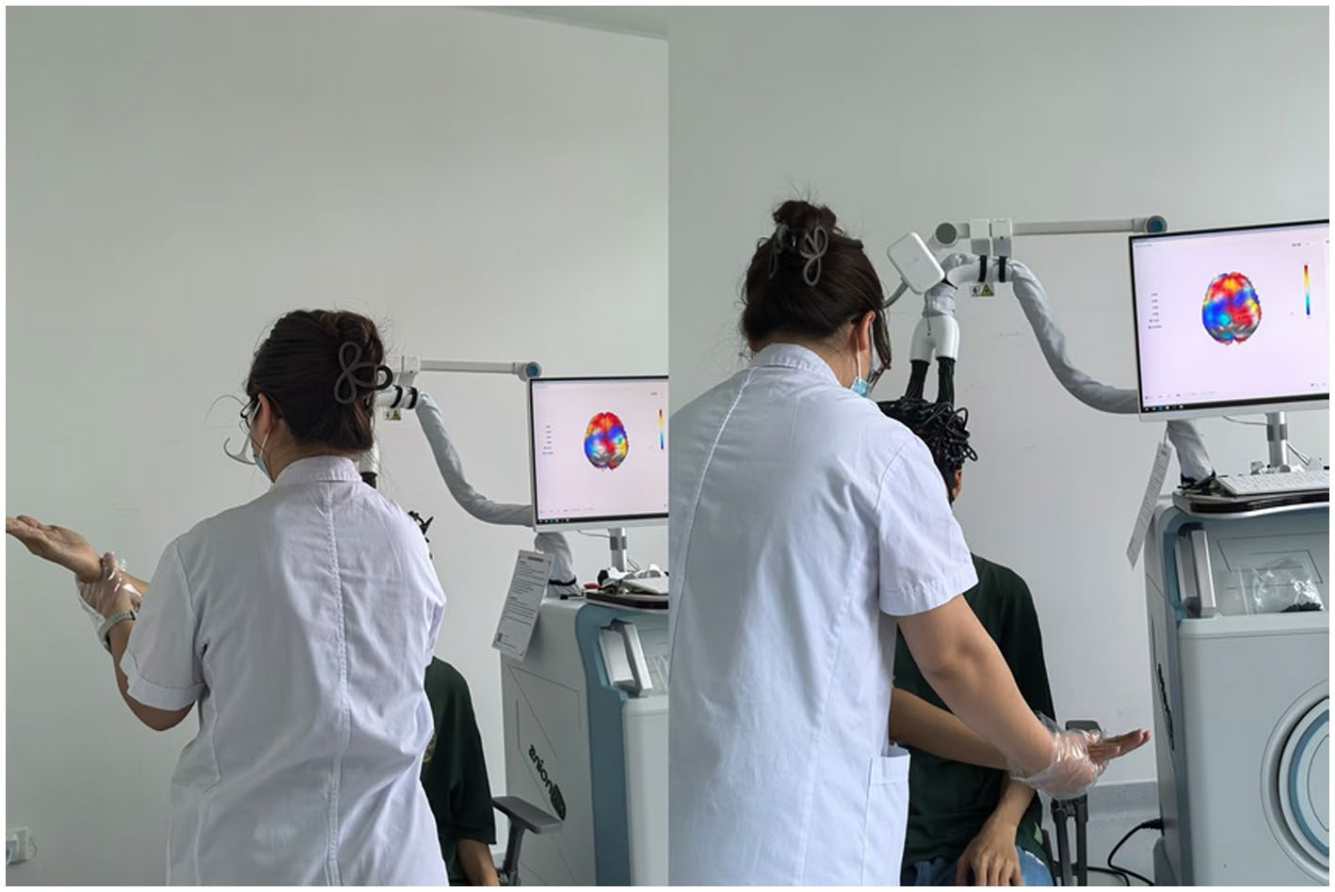
Experimental setup for the PNF D2 flexion task under fNIRS monitoring.

The motor task followed the D2 flexion pattern of Proprioceptive Neuromuscular Facilitation (PNF): starting with the right hand placed laterally to the left knee (thumb upward/outward), participants performed coordinated shoulder (flexion-abduction-external rotation), elbow flexion, and forearm supination at constant velocity under the guidance of a therapist who provided consistent verbal cadence (“flex-2-3, extend-2-3”) for pace control (terminal position: right hand anterior-superior to right shoulder) (20). The movement was performed rhythmically, with one complete flexion-extension cycle executed every 3 s, guided by the therapist’s verbal cues (Figure 2). This resulted in a standardized total of 10 movement cycles per 30-s task block, a protocol that was uniformly applied across all participants and conditions. The timing of task block onsets (at 60s, 120 s, and 180 s) was accurately recorded during the experiment to allow for precise offline marking of the fNIRS data during the preprocessing stage. During AM, participants actively executed PNF movements; during PM, examiners guided movement along identical trajectories; during MI, participants mentally simulated the kinematic chain from a third-person perspective while maintaining physical stillness.

#### 2.2.4 Task selection rationale and visualization

Task selection criteria included: (1) physiological conformity to multi-joint coordination, (2) maximized motor-sensory cortex co-activation (20), and (3) clinical rehabilitation standardization requirements. The selection of this specific PNF pattern was further justified by its capacity to elicit coordinated activation across shoulder, elbow, and forearm musculature, closely mimicking functional reaching movements, which enhances the ecological validity and clinical applicability of our findings.

#### 2.2.5 Protocol standardization

This standardized protocol ensured data reliability through strict control of movement parameters, test intervals and operational procedures, providing a normative experimental paradigm for subsequent brain activation feature analysis.

### 2.3 Data analysis

fNIRS data processing was performed using NirMasterV1.0 (YIRUIDE Medical Co., Wuhan, China) based on MATLAB R2023a (MathWorks, Natick) for preprocessing and analysis. The specific procedures were as follows: (1) Resampling: The data were resampled to a frequency of 20 Hz; (2) Marking: Batch data editing markers were applied at the starting time points of the three task blocks (60s/120s/180 s); (3) Quality control and preprocessing: A comprehensive pipeline was implemented to ensure data quality. First, raw intensity signals were converted to optical density (OD). Data quality was then assessed at both channel and block levels. At the channel level, the coefficient of variation (CV) was calculated for the entire recording duration; channels with CV > 25%, indicating insufficient stability, were excluded. Additionally, channels with an insufficient signal-to-noise ratio (SNR < 20 dB), determined from the cardiac pulsation amplitude in the OD data, were also marked bad. At the block level, any block where more than 50% of channels were identified as bad was entirely discarded. Participant data were rejected if more than 25% of total channels or more than 25% of task blocks were excluded. Following these criteria, a total of 203 out of 1,696 channels (12.0%) across all participants were excluded. No participant met the criteria for overall rejection (>25% bad channels); thus, data from all 16 enrolled participants were included in the final analysis. (4) Preprocessing: Raw signals were converted to optical density data, motion artifacts were corrected using spline interpolation, and a 0.01–0.1 Hz bandpass filter was applied to remove physiological noise from heartbeat (~1 Hz), respiration (~0.2–0.5 Hz), and high-frequency sources. The analysis was performed on a per-epoch basis. Each epoch was time-locked to the onset of a task block, encompassing a 2-s pre-task baseline (rest), the 30-s task period, and the subsequent 28-s post-task period (rest), resulting in a total epoch length of 60 s. This approach of focusing on a 60-s window ensured that the core hemodynamic response evoked by each task could be analyzed independently and robustly; (5) Data analysis: Filtered optical density data were converted to concentration changes of HbO and HbR (*Δ*[HbO] and Δ[HbR]) using the modified Beer–Lambert law, with *Δ*[HbO] selected for statistical analysis due to its higher signal-to-noise ratio and greater sensitivity to cerebral blood flow changes; (6) Final analysis: Three distinct analytical approaches were employed to characterize the brain activation patterns: (a) For spatial activation mapping, a block-averaging method was used. The mean Δ[HbO] concentration during the 0–30s task window was computed for each channel and subjected to one-sample *t*-tests against zero to identify significantly activated brain regions. (b) For activation intensity analysis, a General Linear Model (GLM) was implemented with a canonical hemodynamic response function (HRF) as the regressor of interest. The GLM was fitted to the preprocessed *Δ*[HbO] time series to estimate the beta weights representing the amplitude of the hemodynamic response for each condition in each channel. These beta values, rather than simple mean Δ[HbO] changes, were extracted for statistical analysis. The estimated beta weights were then compared across the three motor therapies using one-way repeated-measures ANOVA followed by *post-hoc* pairwise comparisons where appropriate. (c) For temporal dynamics analysis, two metrics were derived from the block-averaged hemodynamic response: the mean slope during the 2–7 s post-stimulus interval to quantify the initial activation rate, and the T-centroid (center of gravity in the time domain) across the 0–60s window to characterize the temporal distribution of the hemodynamic response. In this context, time zero (t = 0 s) for all temporal analyses was defined as the onset of the therapist’s verbal cue to initiate movement, which closely precedes the actual motor execution and thus captures the very early phase of cortical activation. Both metrics were compared across conditions using one-way repeated-measures ANOVA.

This spatial-intensity-temporal three-dimensional analytical framework systematically reveals the differential neural mechanisms underlying AM, PM, and MI. The statistical inference process for this framework is as follows. We employed multiple comparison correction strategies tailored to different analytical levels. In the spatial activation analysis, the *p*-values from the one-sample *t*-tests conducted on all channels were corrected using the false discovery rate (FDR) method. In the activation intensity and temporal dynamics analyses, the p-values for the main effects from the one-way repeated-measures ANOVAs performed at both the channel and ROI levels were also subjected to FDR correction. All *post-hoc* pairwise comparisons, conducted following a significant main effect in the ANOVA, were performed using paired *t*-tests with Bonferroni correction. Descriptive statistics were applied: continuous variables (e.g., age) underwent normality verification via Shapiro–Wilk tests, with normally distributed data presented as mean ± standard deviation; categorical variables (e.g., gender) were reported as frequencies (percentages).

## 3 Results

### 3.1 Demographic and health characteristics

The study included 16 healthy right-handed participants (8 males, 50.0%; 8 females, 50.0%). Age data followed a normal distribution (Shapiro–Wilk test: W = 0.945, *p* = 0.382) with a mean age of 22.31 ± 1.82 years. All participants had no history of neurological/psychiatric disorders or major somatic diseases, and no adverse events were reported during fNIRS data acquisition.

### 3.2 Comparison of spatial activation patterns across motor therapies

**Figure 3:**
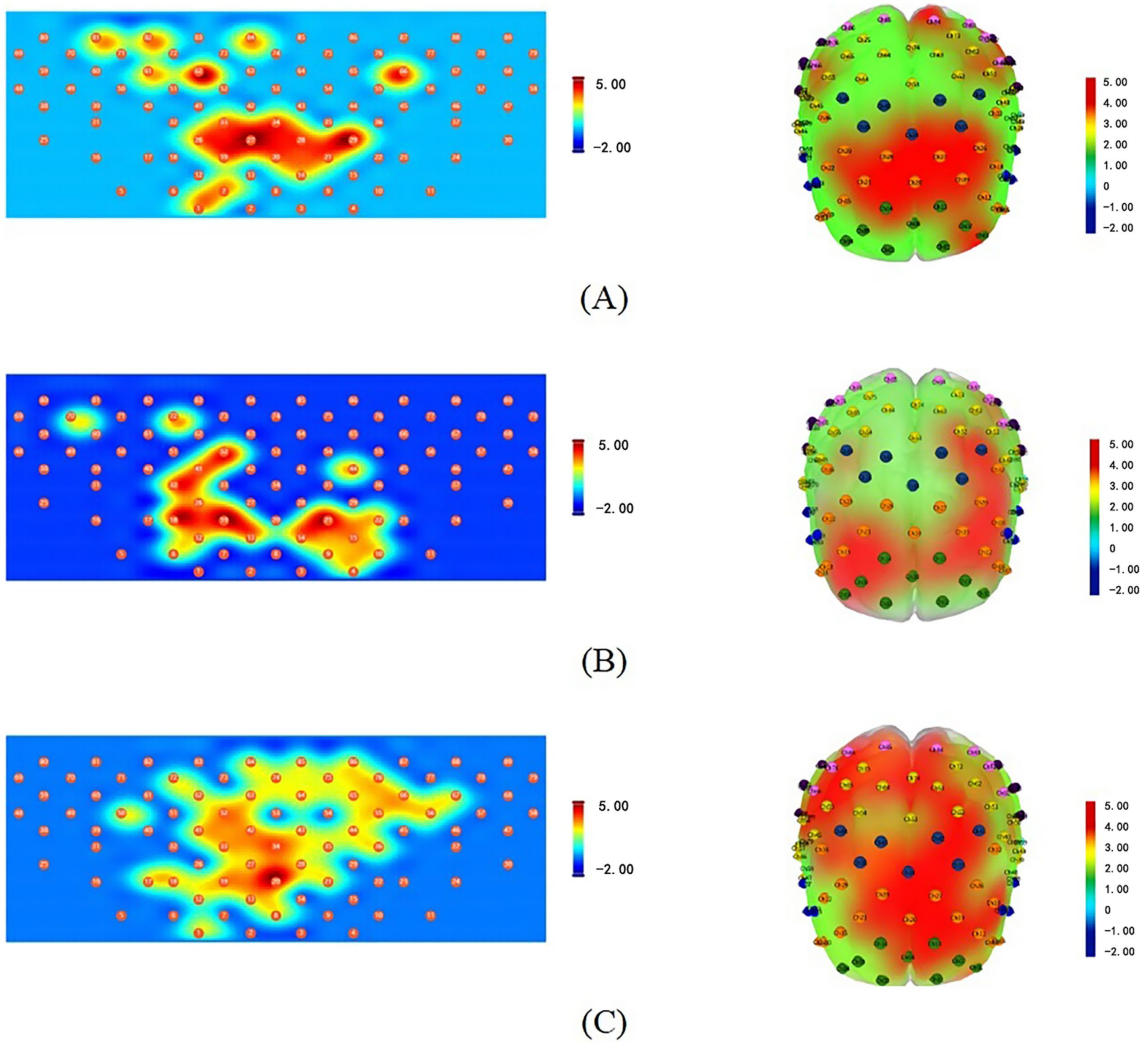
Brain activation topographies of three motor therapies (one-sample *t*-test, FDR-corrected). **(A)** AM: 2D (left) and 3D (right) topographies of *Δ*[HbO] activation; **(B)** PM: 2D (left) and 3D (right) topographies of Δ[HbO] activation; **(C)** MI: 2D (left) and 3D (right) topographies of Δ[HbO] activation. All topographic maps share an identical color scale, with the activation intensity represented on a uniform numerical range from −2 to 5. Red indicates strong positive activation, while blue indicates weak activation or deactivation.

The mean *Δ*[HbO] values during task periods reflect the overall trends in hemoglobin concentration changes. As shown in Figure 3 and Table 1, the three motor therapies exhibited distinct spatial activation patterns. MI demonstrated the most extensive activation (Figure 3C, Table 1), with significant responses in bilateral DLPFC (left: T = 2.53, *p* = 0.038; right: T = 3.08, *p* = 0.015), bilateral SMA (left: *T* = 3.50, *p* = 0.011; right: *T* = 3.82, p = 0.011), bilateral M1 (left: *T* = 2.39, *p* = 0.044; right: T = 3.60, *p* = 0.011), and left FPA (*T* = 3.11, *p* = 0.015). AM showed significant activation in bilateral DLPFC (left: *T* = 4.38, *p* = 0.005; right: *T* = 3.50, *p* = 0.011), bilateral M1 (left: T = 3.96, *p* = 0.006; right: *T* = 2.64, *p* = 0.043), and left FPA (*T* = 2.57, *p* = 0.043) (Figure 3A, Table 1). PM exhibited a more localized activation pattern, with significant responses in bilateral DLPFC (left: *T* = 4.27, *p* = 0.003; right: *T* = 4.43, *p* = 0.003), left S1 (*T* = 2.75, *p* = 0.038), and right FPA (*T* = 3.73, *p* = 0.007) (Figure 3B, Table 1). All results were FDR-corrected.

**Table 1:** One-sample *t*-test results of mean Δ[HbO] during the 0–30s task windows across motor therapies.

ROI	AM (*n* = 16)			PM (*n* = 16)			MI (*n* = 16)		
	(Mean ± SD)	*T*	*P*	(Mean ± SD)	*T*	*P*	(Mean±SD)	*T*	*P*
S1-L	0.010 ± 0.018	2.27	0.064	0.015 ± 0.022	2.75	0.038^**^	0.002 ± 0.032	0.30	0.770
S1-R	0.003 ± 0.019	0.56	0.585	0.004 ± 0.025	0.59	0.561	0.009 ± 0.017	2.12	0.064
M1-L	0.023 ± 0.023	3.96	0.006^**^	0.013 ± 0.023	2.21	0.072	0.013 ± 0.022	2.39	0.044^**^
M1-R	0.017 ± 0.026	2.64	0.043^**^	0.014 ± 0.035	1.55	0.178	0.019 ± 0.021	3.60	0.011^**^
DLPFC-L	0.017 ± 0.015	4.38	0.005^**^	0.017 ± 0.016	4.27	0.003^**^	0.009 ± 0.015	2.53	0.038^**^
DLPFC-R	0.015 ± 0.017	3.50	0.011^**^	0.016 ± 0.014	4.43	0.003^**^	0.009 ± 0.011	3.08	0.015^**^
SMA-L	0.011 ± 0.020	2.17	0.066	0.010 ± 0.020	2.07	0.081	0.010 ± 0.012	3.50	0.011^**^
SMA-R	0.006 ± 0.012	1.96	0.086	0.006 ± 0.019	1.17	0.289	0.009 ± 0.010	3.82	0.011^**^
FPA-L	0.019 ± 0.029	2.57	0.043^**^	0.019 ± 0.031	2.43	0.057	0.015 ± 0.019	3.11	0.015^**^
FPA-R	0.009 ± 0.028	1.28	0.244	0.023 ± 0.024	3.73	0.007^**^	0.009 ± 0.017	2.06	0.064

Data are expressed as mean ± standard deviation. One-sample *t*-tests were performed to compare the mean Δ[HbO] during the task period against a baseline of zero for each region of interest. *T*-values from the *t*-tests are reported. ^*^p < 0.05 (uncorrected), ^**^p < 0.05 (FDR-corrected for multiple comparisons across channels). ROI, region of interest.

### 3.3 Comparison of activation intensity across motor therapies

**Figure 4:**
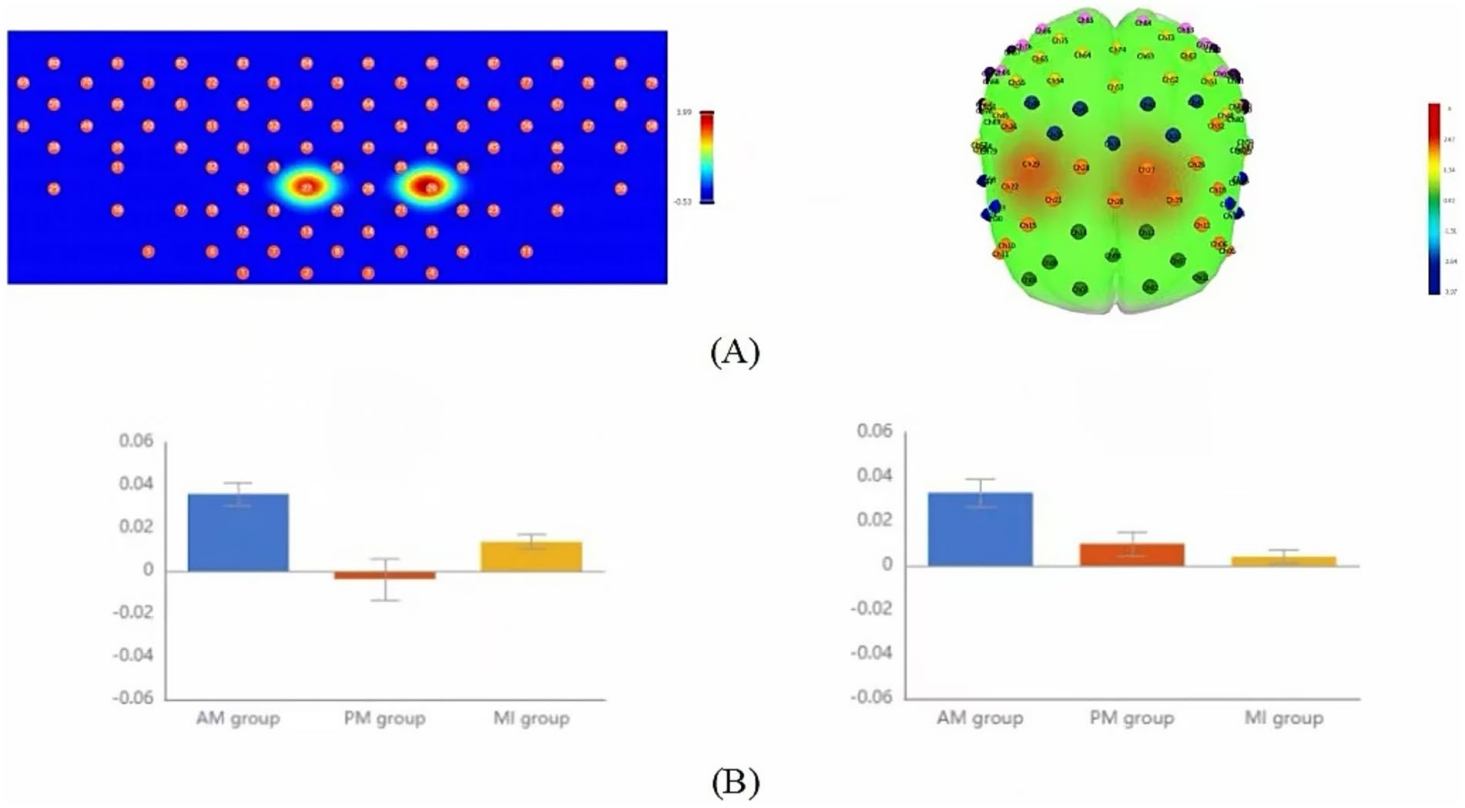
Comparison of GLM mean activation features among the three motor therapies. **(A)** Topographic maps (2D, left; 3D, right) showing brain regions with significant differences in activation intensity across the AM, PM, and MI conditions based on one-way repeated-measures ANOVA (*p* < 0.05, uncorrected); **(B)** Bar plots comparing GLM means of channels exhibiting significant between-group differences: left DLPFC channel 27 (CH27, left) and right DLPFC channel 28 (CH28, right). Color scale: Red indicates strong activation, blue indicates weak activation. Error bars represent standard error.

In fNIRS data analysis, the GLM-fitted beta weights served as the key indicator for assessing regional activation intensity. Although one-way repeated measures ANOVA revealed no significant differences at the whole region-of-interest level, significant between-group differences were observed in specific channels (Figure 4A, Table 2). Specifically, channel 27 in the left DLPFC showed significant activation intensity differences among the three therapies (*F* = 3.65, *p* = 0.038, uncorrected). *Post-hoc* multiple comparisons demonstrated that AM yielded significantly higher significantly higher GLM beta weights than both PM and MI. Similarly, channel 29 in the right DLPFC exhibited significant between-group differences (*F* = 3.99, *p* = 0.029, uncorrected), with *post-hoc* tests indicating significantly greater GLM-estimated response amplitudes in AM compared to MI, though the AM-PM comparison did not reach statistical significance. These findings collectively suggest that AM demonstrates relatively superior activation characteristics in bilateral DLPFC channels (Figure 4B, Table 2).

**Table 2:** One-way repeated measures ANOVA for channels with significant activation differences in GLM means.

CH	AM (*n* = 16) (Mean ± SD)	PM (*n* = 16) (Mean ± SD)	MI (*n* = 16) (Mean ± SD)	*F*	*P*	*Post-hoc* tests
GLM-CH27	0.036 ± 0.030	−0.004 ± 0.053	0.014 ± 0.019	3.65	0.038*	AM>PM, MI
GLM-CH29	0.033 ± 0.035	0.010 ± 0.031	0.003 ± 0.017	3.99	0.029*	AM>MI
Slope-CH27	0.0013 ± 0.001	0.0002 ± 0.001	0.0002 ± 0.001	10.31	0.034^**^	AM>PM, MI

Data are presented as mean ± standard deviation. CH, channel; GLM, General Linear Model. *F*-value represents the statistic from one-way repeated measures ANOVA. ^*^p < 0.05 (uncorrected), ^**^p < 0.05 (FDR corrected for multiple comparisons). *Post-hoc* analyses were conducted using Bonferroni-corrected paired *t*-tests following a significant main effect.

### 3.4 Comparison of temporal dynamics across motor therapies

**Figure 5:**
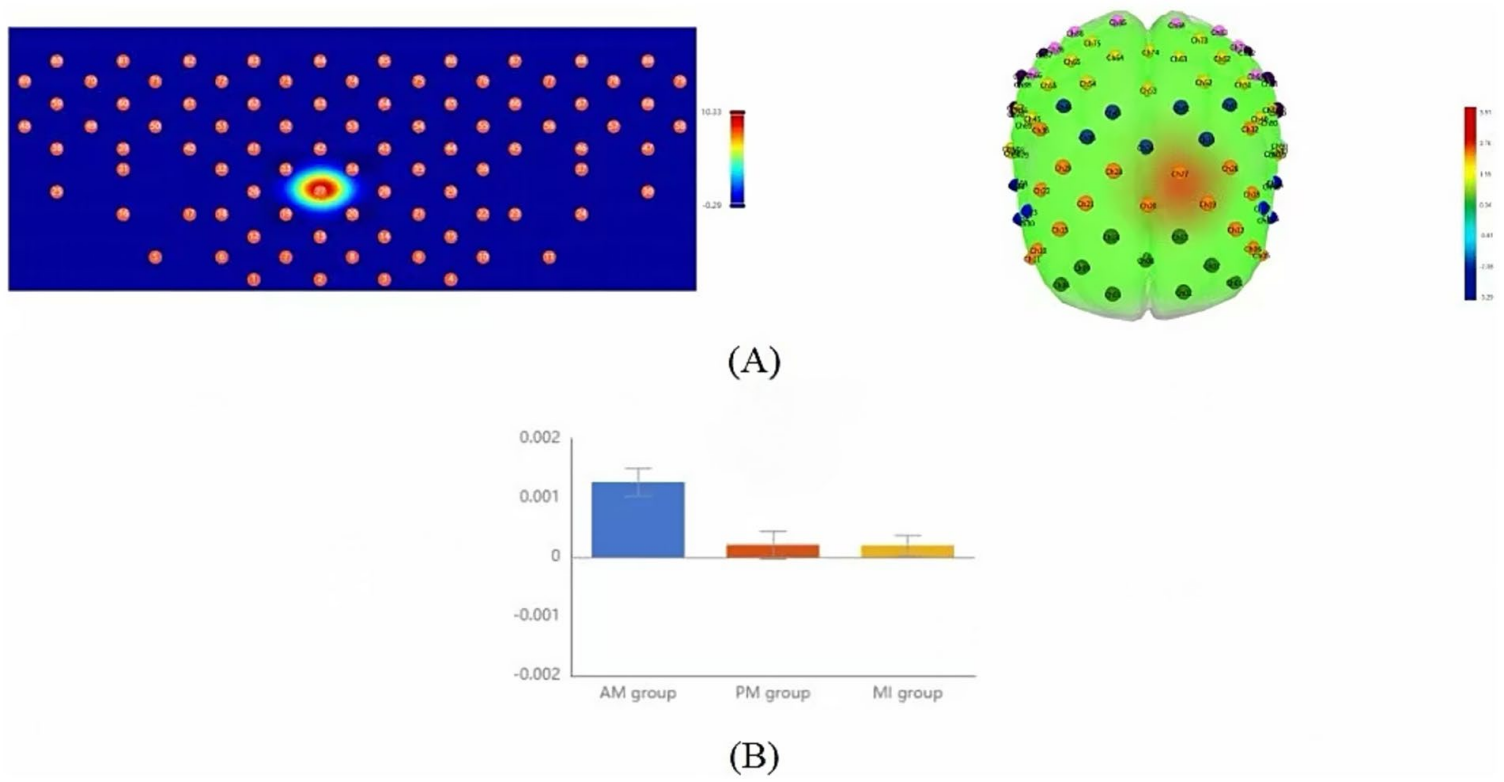
Comparison of slope mean activation features among the three motor therapies. **(A)** Topographic maps (2D, left; 3D, right) showing brain regions with significant differences in the initial activation rate (slope) across the AM, PM, and MI conditions based on one-way repeated-measures ANOVA (*p* < 0.05, FDR-corrected). **(B)** Bar plot comparing slope means of left DLPFC channel 27 (CH27) exhibiting significant between-group differences. Color scale: Red indicates strong activation, and blue indicates weak activation. Error bars represent standard error.

In fNIRS data, the slope parameter reflects the rate of *Δ*[HbO] concentration changes during tasks and can be used to evaluate the rapidity of brain activation. One-way repeated measures ANOVA showed significant between-group differences in specific channels (Figure 5A, Table 3). Specifically, channel 27 in the left DLPFC showed significant differences in mean slope values among the three therapies (*F* = 10.31, *p* = 0.034, FDR-corrected). *Post-hoc* multiple comparisons showed AM had a significantly higher slope than PM and MI (Figure 5B, Table 3).

**Table 3:** One-way repeated measures ANOVA for channels with significant activation differences in slope means.

CH	AM (*n* = 16) (Mean ± SD)	PM(*n* = 16) (Mean ± SD)	MI (*n* = 16) (Mean ± SD)	*F*	*P*	*Post-hoc* tests
Slope-CH27	0.0013 ± 0.001	0.0002 ± 0.001	0.0002 ± 0.001	10.31	0.034^**^	AM>PM, MI

Data are presented as mean ± standard deviation. CH, channel; Slope, the mean slope of Δ[HbO] during the 2–7 s post-stimulus interval. *F*-value represents the statistic from one-way repeated measures ANOVA. *p < 0.05 (uncorrected), ^**^p < 0.05 (FDR corrected for multiple comparisons). *Post-hoc* analyses were conducted using Bonferroni-corrected paired *t*-tests following a significant main effect.

**Figure 6:**
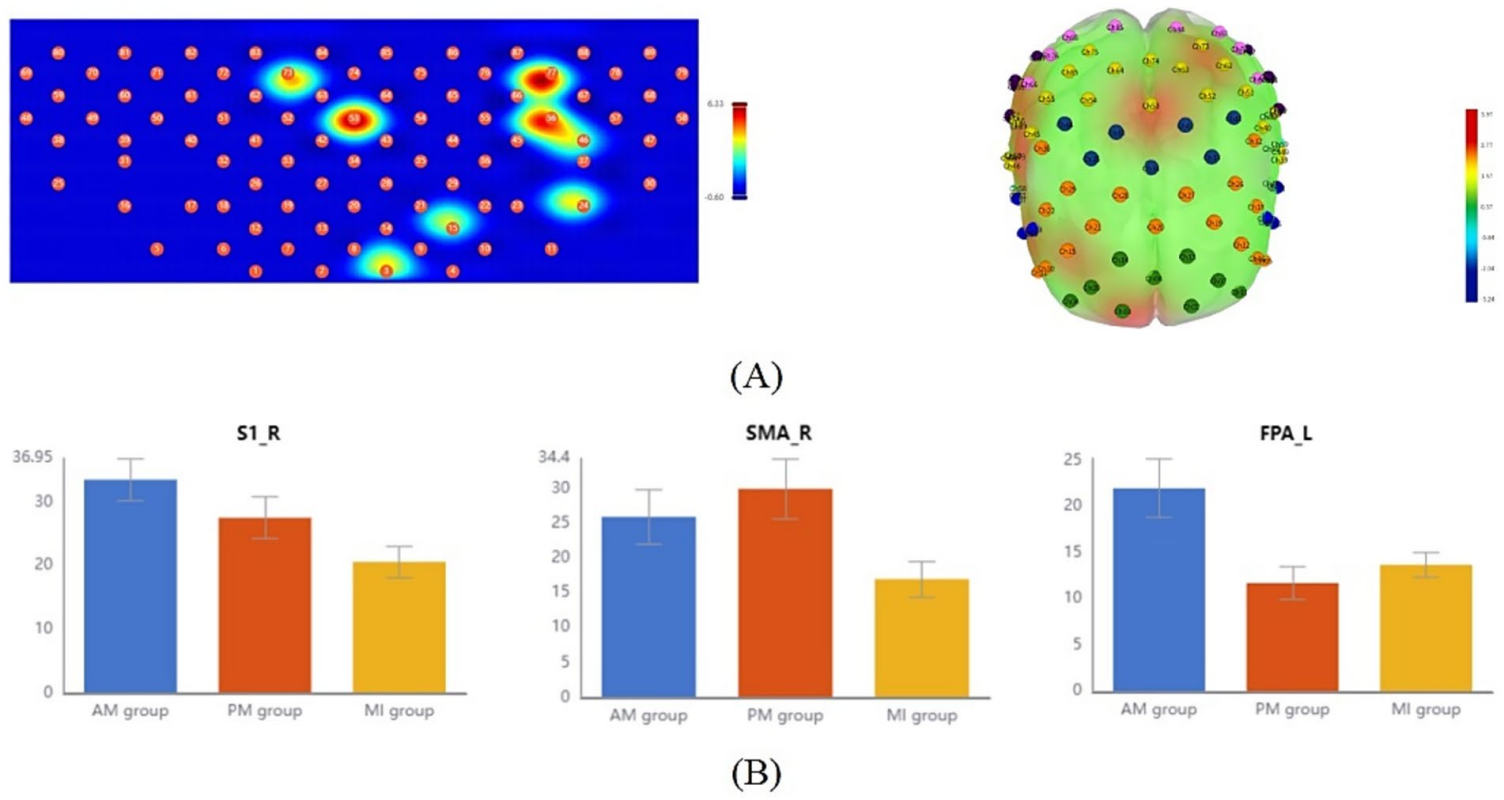
Comparison of T-centroid mean activation features among the three motor therapies. **(A)** Topographic maps (2D, left; 3D, right) showing brain regions with significant differences in the temporal distribution of the hemodynamic response (T-centroid) across the AM, PM, and MI conditions based on one-way repeated-measures ANOVA (*p* < 0.05, FDR-corrected). **(B)** Bar plots comparing T-centroid means of regions of interest (ROIs) with significant between-group differences: from left to right are S1-R, SMA-R, and FPA-L. Color scale: Red indicates strong activation, and blue indicates weak activation. Error bars represent standard error.

The T-centroid refers to the time-weighted average of hemoglobin concentration change curves during tasks, reflecting the temporal distribution characteristics of signal changes, where smaller T-centroid values indicate faster brain activation responses to movement. One-way repeated measures ANOVA showed significant between-group differences in specific ROIs (Figure 6A, Table 4). Specifically, significant differences in mean T-centroid were found in the right SMA (*F* = 5.42, *p* = 0.03, FDR-corrected) and right S1 (*F* = 6.12, p = 0.03, FDR-corrected), with *post-hoc* tests showing MI had significantly earlier response times than both AM and PM. Finally, left FPA also showed significant differences in mean T-centroid (*F* = 7.00, p = 0.03, FDR-corrected), with both PM and MI showing faster responses than AM in this region (Figure 6B, Table 4).

**Table 4:** One-way repeated measures ANOVA for region area of interest with significant activation differences in T center of gravity means.

ROI	AM (*n* = 16) (Mean ± SD)	PM (*n* = 16) (Mean ± SD)	MI (*n* = 16) (Mean ± SD)	*F*	*P*	*Post-hoc* tests
S1-L	25.02 ± 12.18	22.43 ± 12.86	20.62 ± 10.02	0.57	0.82	\
S1-R	33.58 ± 13.09	27.58 ± 13.31	20.62 ± 9.542	6.12	0.03^**^	AM>MI
M1-L	18.17 ± 10.69	17.12 ± 7.824	17.39 ± 11.14	0.05	0.95	\
M1-R	20.23 ± 13.62	25.27 ± 15.05	19.38 ± 11.26	1.04	0.69	\
DLPFC-L	19.96 ± 14.13	13.93 ± 7.252	17.03 ± 11.87	1.23	0.69	\
DLPFC-R	16.89 ± 9.628	10.54 ± 10.46	17.55 ± 10.51	0.19	0.95	\
SMA-L	24.32 ± 13.85	19.16 ± 9.476	21.43 ± 13.42	0.91	0.69	\
SMA-R	25.99 ± 15.43	30.02 ± 17.13	17.02 ± 10.29	5.42	0.03^**^	PM, AM>MI
FPA-L	22.00 ± 12.63	11.74 ± 7.151	13.72 ± 5.306	7.00	0.03^**^	AM>PM, MI
FPA-R	19.33 ± 10.45	19.17 ± 12.26	17.71 ± 11.74	0.10	0.95	\

Data are presented as mean ± standard deviation. T-centroid represents the temporal center of gravity of the Δ[HbO] signal during the 0–60s task window. ROI, region of interest. *F*-value represents the statistic from one-way repeated measures ANOVA. *p < 0.05 (uncorrected), ^**^p < 0.05 (FDR corrected for multiple comparisons). *Post-hoc* analyses were conducted using Bonferroni-corrected paired *t*-tests following a significant main effect.

## 4 Discussion

Spatially, the three motor therapies demonstrated distinct brain activation patterns. MI exhibited the most extensive activation, encompassing both the motor execution network (bilateral SMA and M1) and higher-order cognitive control regions (bilateral DLPFC and left FPA). AM showed characteristic motor execution network activation, primarily involving bilateral DLPFC, bilateral M1, and left FPA, while PM displayed more limited activation in bilateral DLPFC, left S1, and right FPA. Notably, our study revealed similar activation patterns between AM and MI in healthy adults, with shared activation in bilateral DLPFC, bilateral M1, and left FPA. These findings align with studies by Chen et al. (21) and Zhang et al. (22), demonstrating that MI can activate neural networks similar to AM. Chholak et al. (23) further confirmed that MI and motor execution share comparable neural mechanisms, both eliciting movement-related event-related desynchronization (ERD). This indicates that motor imagery can simulate actual movement in terms of brain activation patterns, providing critical neuroimaging evidence for MI’s therapeutic mechanisms. Within bilateral M1, significant activation was observed not only during AM but also during MI. This phenomenon can be explained by functional subdivisions of M1. Researchers have divided M1 into two subregions: the anterior region (BA4a), adjacent to premotor and supplementary motor areas (BA6), is primarily activated during motor imagery, while the posterior region (BA4p) is more strongly associated with pure motor execution (24, 25). This suggests that M1’s functions extend beyond motor execution to include advanced processes like motor planning and control. However, it’s important to note that MI and motor execution do not activate identical brain networks. For instance, our study found additional SMA activation during MI compared to AM, supporting Stephan et al.’s (26) proposition that SMA plays a crucial role in motor imagery due to its involvement in movement planning.

In bilateral FPA, our study revealed distinct activation patterns: AM and MI primarily engaged left FPA, whereas PM predominantly activated right FPA. This lateralization likely reflects specialized neural pathways—the left FPA, as a key node in the dorsolateral prefrontal network, plays a pivotal role in motor learning (particularly sequential action encoding) (27). Both AM’s voluntary movement planning and MI’s mental simulation require left FPA involvement in action sequence construction, consistent with fNIRS studies demonstrating left prefrontal dominance during complex motor tasks (28). In contrast, right FPA activation may relate to attentional resource allocation. As PM relies on therapist/device guidance requiring continuous monitoring of external cues, the right prefrontal cortex—established as dominant for sustaining alertness and spatial attention (29)—becomes preferentially engaged. This aligns with fNIRS evidence showing positive correlations between right FPA hemodynamic responses and attentional focus during passive training (30). Additionally, PM uniquely activated left S1, corroborating previous fNIRS and fMRI studies reporting examiner’s S1 activation during PM administration, particularly in contralateral S1 (31). This further confirms that PM’s externally driven joint mobilization inherently enhances somatosensory input regarding limb position and movement velocity.

In terms of activation intensity, this study revealed significantly stronger GLM-estimated hemodynamic responses in bilateral DLPFC channels during AM compared to other conditions. As a critical hub mediating advanced motor control functions through its diverse anatomical projections to motor regions (32), the DLPFC has been extensively documented to participate in higher cognitive processes, including executive function (33), response selection (34), response initiation (35), and response inhibition (36). Our finding of significant bilateral DLPFC activation across all three motor therapies further underscores its pivotal role in motor rehabilitation. Additionally, the DLPFC contributes to motor attention and working memory. Our results suggest that the DLPFC may be particularly engaged in tasks requiring cognitive flexibility—the ability to adapt attentional focus and behavioral responses according to task demands—which explains its greater involvement during AM versus PM and MI. This differential engagement may also stem from Smidt et al. (1) the absence of actual movement in MI reducing DLPFC demands for sensorimotor integration and (2) the externally driven nature of PM decreasing reliance on DLPFC-mediated executive control.

Temporal dynamics analysis revealed distinct timing characteristics of brain activation across motor therapies. Slope analysis demonstrated significantly faster activation rates in the left DLPFC (channel 27) during AM compared to PM and MI, aligning with Miller and Cohen’s (37) executive control theory of DLPFC. This suggests that AM’s self-generated motor intentions enable quicker mobilization of DLPFC cognitive control resources, thereby accelerating neural encoding of movement plans. T-centroid analysis further showed significantly earlier response times in the right SMA and right S1 during MI versus AM and PM, supporting Jeannerod’s (38) motor simulation theory that mental imagery bypasses physiological delays of actual execution through direct activation of premotor networks via internal action representations. Notably, Hardwick et al. (39) confirmed that motor imagery elicits early motor cortex activation patterns similar to actual movement but with superior temporal efficiency. In the left FPA, both PM and MI showed faster responses than AM, potentially reflecting distinct neural mechanisms: PM’s externally driven nature reduces cognitive load in motor decision-making, while MI optimizes action representation efficiency through mental simulation. In contrast, AM requires full motor planning-execution integration, resulting in slower responses due to more complex neural processing. These findings reveal fundamental differences in prefrontal information-processing efficiency across therapies.

Clinically, the neural mechanisms revealed by our fNIRS-based multidimensional analysis provide new insights for optimizing rehabilitation strategies and pave the way for novel clinical applications. In contrast to the traditional view that primarily applies MI in the early rehabilitation stage (40), our study found that MI broadly activates the sensorimotor network and prefrontal cognitive control regions. This whole-brain network co-activation pattern suggests that MI may not only be suitable for patients with early-stage motor deficits, but its potential to promote cross-network neuroplasticity could make it an important intervention throughout the entire rehabilitation process. Furthermore, the distinct cortical activation maps we identified could potentially serve as objective neuroimaging biomarkers to monitor therapy progress and tailor rehabilitation protocols based on individual patients’ neural engagement patterns. Compared to previous studies focusing mainly on the role of AM in enhancing muscle strength and improving joint mobility (41), our study is the first to reveal, from multiple dimensions, the unique advantages of AM in activating the DLPFC and accelerating neural responses. This provides a new neuroscientific basis for its clinical application in improving motor execution function and facilitating motor learning. The robust and rapid DLPFC activation characteristic of AM makes it a particularly suitable candidate for integration with neurofeedback techniques, potentially enhancing cognitive engagement and motor learning outcomes during therapy. Of particular note, while PM has long been considered an auxiliary training method for maintaining joint range of motion (42), our fNIRS findings demonstrate its unique sensory-attentional network activation pattern. This not only confirms its traditional value but, more importantly, reveals its independent rehabilitative value in promoting sensory integration and attention training. This finding suggests that PM could be strategically employed in patients with attentional deficits or sensory neglect, and fNIRS could offer a potential means to monitor its effectiveness by tracking the normalization of sensory-attentional network activation.

However, this study has limitations. First, while each motor therapy condition was tested across three separate task blocks, providing a more robust basis for analysis than a single trial, future studies could potentially enhance the signal stability and statistical power by incorporating a higher number of repetitions, as is common practice in block-design fNIRS studies. Second, the sample size was relatively small and consisted solely of healthy, right-handed young adults, which may restrict comprehensive characterization of therapy-specific activation patterns and limit the generalizability of the findings to clinical populations (e.g., stroke patients). Future studies should explore the effects of a greater number of task repetitions, expand sample sizes, and include neurological patients to validate clinical applicability and provide more direct neuroimaging evidence for rehabilitation practice. Third, due to the constraints of our current fNIRS data analysis pipeline, the comparisons of activation intensity and temporal dynamics were conducted at the single-channel level rather than averaging across regions of interest (ROIs). While reporting significant findings from individual channels is a common practice in exploratory fNIRS studies and can reveal localized ‘hotspots’ of neural activity (43, 44), it may not fully represent the hemodynamic response of an entire functional brain area. Future studies should incorporate ROI-based averaging techniques to enhance the robustness and generalizability of the findings.

## 5 Conclusion

This study employed fNIRS-based multidimensional analysis to elucidate distinct neural mechanisms underlying AM, PM, and MI. Spatially, MI broadly activated a motor-cognitive integration network through mental simulation, AM primarily reinforced the motor execution network, while PM predominantly engaged the sensory-attentional network. In terms of activation intensity, AM demonstrated significant dominance in prefrontal control regions. Temporally, AM exhibited rapid initiation advantages, MI showed efficient neural response properties, and PM optimized sensory information processing. By integrating fNIRS multidimensional analytics with clinical rehabilitation needs, this work (1) establishes a novel paradigm for investigating motor therapy mechanisms, (2) provides innovative technological pathways and theoretical frameworks for precision rehabilitation systems, and (3) offers scientific foundations for developing neuroimaging biomarker-guided personalized rehabilitation protocols.

## Data Availability

The raw data supporting the conclusions of this article will be made available by the authors, without undue reservation.
